# All-lignocellulosic Fiberboard from Steam Exploded *Arundo Donax* L.

**DOI:** 10.3390/molecules23092088

**Published:** 2018-08-21

**Authors:** Diego Ramos, Nour-Eddine El Mansouri, Francesc Ferrando, Joan Salvadó

**Affiliations:** 1 Department of Mechanical Engineering, Rovira i Virgili University, Avinguda dels Països Catalans 26, 43007 Tarragona, Spain; diego.ramos@urv.cat (D.R.); f.ferrando@urv.cat (F.F.); 2 Department of Chemistry, Faculté Polydisciplinaire, Sultan Moulay Slimane University, Mghila BP.592, Béni-Mellal 23000, Morocco; 3 Laboratory of Biological Engineering, Faculté des Sciences et Techniques, Sultan Moulay Slimane University, Mghila BP.523, Béni-Mellal 23000, Morocco; 4 Department of Chemical Engineering, Rovira i Virgili University, Avinguda dels Països Catalans 26, 43007 Tarragona, Spain

**Keywords:** all-lignocellulosic, binderless, fiberboards, *Arundo donax* L., steam explosion, water resistance, mechanical properties

## Abstract

This paper explores the possibility of producing all-lignocellulosic fiberboards from *Arundo donax* L. as a source of lignocellulosic fibers with no synthetic binders. This raw material was steam exploded with a thermomechanical aqueous vapor process in a batch reactor. The *Arundo donax* raw material and its obtained pulp were characterized in terms of chemical composition and the results were compared to other lignocellulosic materials. The chemical composition of steam exploded *Arundo* fibers showed high cellulose and a moderate lignin content suggesting it was a good raw material for fiberboard production. The all-lignocellulosic fiberboards were produced on laboratory scale; using the steam exploded *Arundo donax* by means of a wet process. The effects of pressing pressure on physical and mechanical properties were evaluated and the conditions that optimize the responses were found. The analyzed properties were density (d); water absorption (WA); thickness swelling (TS); modulus of elasticity (MOE); modulus of rupture (MOR); and internal bond strength (IB). The tested levels of the pressing pressure range from 0.35 to 15 MPa. The optimum IB; MOE; MOR; WA and TS were 1.28 MPa, 7439 MPa, 40.4 MPa, 17.6% and 13.3%, respectively. The obtained fiberboards were of very good quality and more than satisfy the requirements of the relevant standard specifications.

## 1. Introduction

*Arundo donax* L., also known as giant reed, is a cane species considered as an important source of biomass because of its high biomass production and rapid growth. Other advantages of this plant are the low agronomic input, low production costs, and the ability to grow in different kinds of environments [1,2]. In addition, the *Arundo* biomass has higher content in fibrous materials with interesting morphological and chemical characteristics [3,4], suitable for its application in ethanol production and also as a source of pulp for papermaking [5,6,7]. However, there is little research on its application in fiber-based composites, with the exception of a recently published study exploring fibers from steam exploded *Arundo* biomass for high performance fiberboards [8].

Researchers have been studying different ways of using various lignocellulosic materials for fiberboard production. In our research group, steam explosion treatment has been extensively studied [8,9,10,11,12,13]. This technique is considered one of the best ways for pre-treating lignocellulosic materials used for fiberboards and biocomposites. This is because steam explosion pre-treatment can preserve the fiber structure and separate the lignocellulosic material into its main components [10]. In addition, the obtained fibers are well separated and the lignin can easily exude from the cell wall to the fiber surface [9]. The lignin thermo-plasticity is an important property in promoting the thermal adhesion of the fibers, from which the board is formed by means of hot pressing [14]. This paper explores the possibility of producing all-lignocellulosic fiberboard from steam exploded *Arundo donax* L. as a source of fibers with no synthetic binders. It deals with the effects of the pressing pressure on the physical and mechanical properties of all-lignocellulosic fiberboards.

## 2. Results and Discussion

### 2.1. Chemical Composition of Arundo Donax Raw Material and Pulp

The chemical composition of *Arundo donax* was determined according to the protocols mentioned in the Materials and Methods section. The results shown in Table 1 were compared to non-wood and wood lignocellulosic materials used in fiberboards from the literature [2,6,12,15,16]. The chemical composition results coincide with those reported by other investigators for *Arundo* biomass [2,6]. This raw material had a slightly higher lignin content compared to *Miscanthus sinensis* and *Eucalyptus globulus*, but lower than *Vitis vinifera* and *Pine pinaster*. Cellulose accounted for 43.1%, a value slightly higher than those reported in literature for *Arundo* biomass [2,6]. In addition, the cellulose content was lower than of pine and eucalyptus wood, but similar to that of *Miscanthus sinensis* and *Vitis vinifera*. The hemicellulose content was lower than *Eucalyptus globulus* and higher than *Pine pinaster*; but similar to that of *Miscanthus sinensis* and *Vitis vinifera*. Regarding the ash content, which is mainly composed of silicates and mineral components (Na, Fe, Mn, K, etc.), it was higher than *Miscanthus sinensis*, *Pine pinaster* and *Eucalyptus globulus*, but in the same degree of *Vitis vinifera*. The amount of extractives was lower than that of *Vitis vinifera* and higher that *Miscanthus sinensis*. The holocellulose is the moiety of cellulose and hemicellulose in the fibers. The holocellulose was lower than pine and eucalyptus wood, but slightly higher than *Vitis vinifera*. From this comparison, it can be concluded that *Arundo* biomass has medium cellulose and high lignin percentages, suggesting it as a suitable alternative source of fibers from wood and non-wood lignocellulosic materials used in board production. 

Afterwards, *Arundo donax* raw material was submitted to steam explosion treatment according to the reported experimental procedure. This treatment significantly affected the chemical composition, since cellulose and lignin were increased in the remaining material, while the extractives, ashes, and hemicelluloses were decreased, as can be seen in Table 1. 

These results are in agreement with those reported for wheat straw and Miscanthus sinensis [12,17]. In addition, similar results to ours with regards to the behaviour of cellulose, hemicelluloses, and lignin have been obtained for other materials [13,18,19]. According to some authors the low hemicelluloses content is responsible for improving the board’s water resistance [12,13]. Moreover, the reduction of hemicellulose and extractives are responsible for the increase in crystallinity of the fibrous material [20], a fact that should be reflected in the mechanical properties of fiberboards.

### 2.2. Effects on Physical Properties

**Figure 1:**
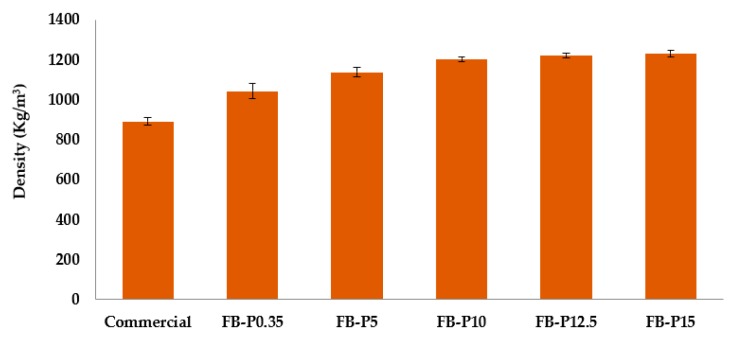
Mean density (d) of the produced all-lignocellulosic fiberboards.

**Figure 2:**
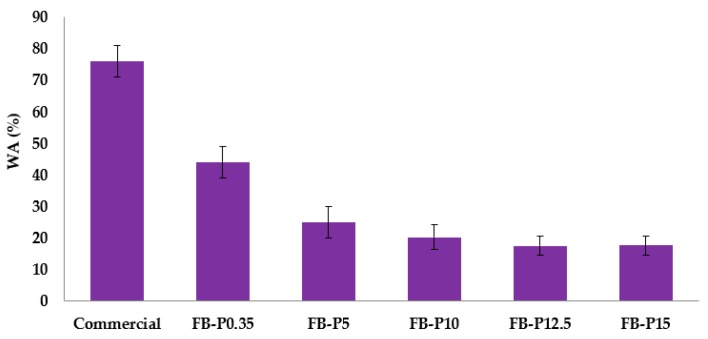
Mean water absorption (WA) of the produced all-lignocellulosic fiberboards.

**Figure 3:**
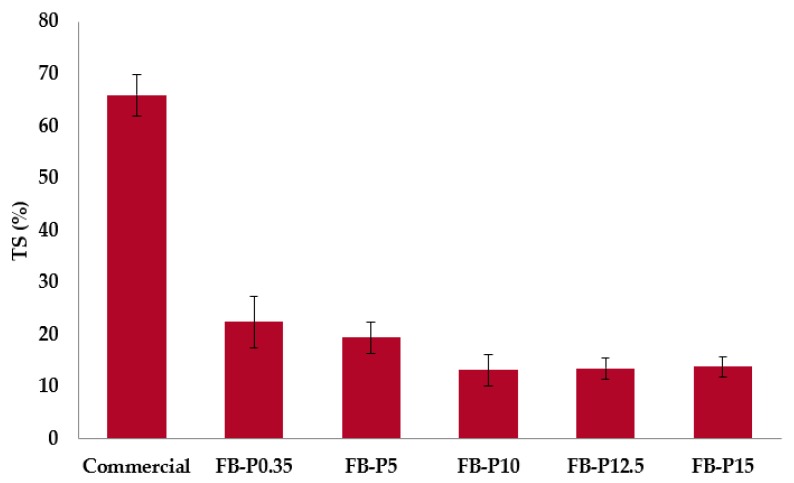
Mean thickness swelling (TS) of the produced all-lignocellulosic fiberboards.

Table 2 summarizes the physical properties of various all-lignocellulosic fiberboards prepared at different pressing pressure, also illustrated in Figure 1, Figure 2 and Figure 3. The effect of pressing pressure on the board’s density was studied, and Figure 1 shows the evolution of this property as a function of pressing pressure, compared to that of the commercial board. A strong increase in density is observed during the first 10 MPa pressing pressure. These increases were 16.7%, 27.5%, and 34.6% for pressing pressure of 0.35, 5 and 10 MPa, respectively. A further increase in pressing pressure leads to a slight increase, reaching a maximum density at 15 MPa (*ρ*_15 MPa_ = 1231 MPa). Therefore, the obtained all-lignocellulosic fiberboards at the maximum pressing pressure present fewer voids. In fact, for the same fibers weight, fiberboard pressed at higher pressing pressure can hold less volume, and consequently the density is increased. 

The water absorption (WA) and thickness swelling (TS) were measured according to the European standard (EN317). They are defined as the percentage increase in the thickness and weight of fiberboard, after immersing in water for 24 h at room temperature. They were measured to assess the water resistance, and the results are shown in Figure 2 and Figure 3. Overall, the obtained all-lignocellulosic fiberboards with steam exploded *Arundo donax* fibers absorbed less water compared to the commercial board. This was evident from the results of the fiberboards made even at lower pressing pressure (0.35 MPa), which exhibit lower water absorption properties compared to the commercial board. In addition, as can be shown in Figure 2, the water absorption of the produced fiberboards decreased with the increase of the applied pressing pressure, reaching minimal values of 17.6% and 17.7% for a pressing pressure of 12.5 and 15 MPa, respectively. This trend confirmed that pressing pressure had a positive effect on the water resistance. 

The WA and TS were measured using the same experimental setup and both properties are directly related to each other. Consequently, similar trends were observed as can be shown in Figure 3. The TS of fiberboards based on steam exploded fibers reached a minimal value of 13.3% for boards pressed at 10 MPa. According to the European standard (EN317), the TS and WA for wood panels should be lower than 20% and 30%, respectively. Overall, all fiberboards prepared following the reported experimental procedure complied with these requirements for the pressing pressure over 5 MPa. 

The improvement in the fiberboard’s dimensional stability is mainly attributed to the high hydrophobicity of steam-exploded *Arundo donax* fibers. This hydrophobicity is due to the steam explosion effect since it can reduce extractives and hemicelluloses contents of the raw material, and the lignin can easily exude from the cell wall to the fiber surface [9]. Therefore, the lower extractives and hemicelluloses content prevented water from entering into the fiber’s molecular chains and consequently improved the water resistance of the boards [21]. This improvement in water resistance can also be explained by the fact that the cell wall is bulked by lignin; this makes it hydrophobic and water cannot enter and swell up the cell wall. In addition, the non-polar hydro-carbon chains and aromatic rings in the lignin molecule at fiber surface are able to improve the water resistance of fiberboards [16,22]. The same effect was observed when adding exogenous Kraft lignin as green adhesive in fiberboards made from other agricultural wastes [16,23]. 

### 2.3. Effects on Mechanical Properties

**Figure 4:**
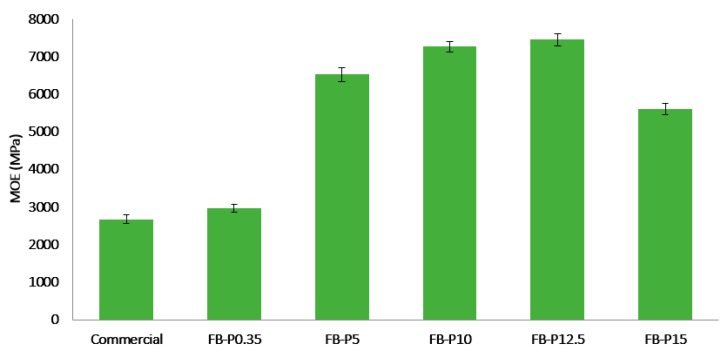
Mean modulus of elasticity (MOE) of the produced all-lignocellulosic fiberboards.

**Figure 5:**
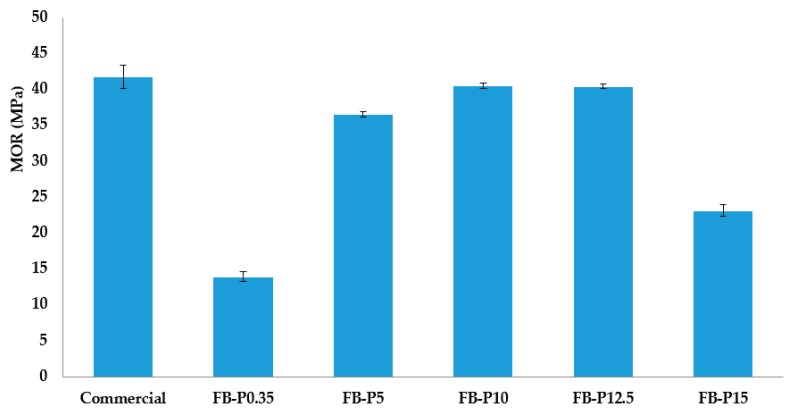
Mean modulus of rupture (MOR) of the produced all-lignocellulosic fiberboards.

**Figure 6:**
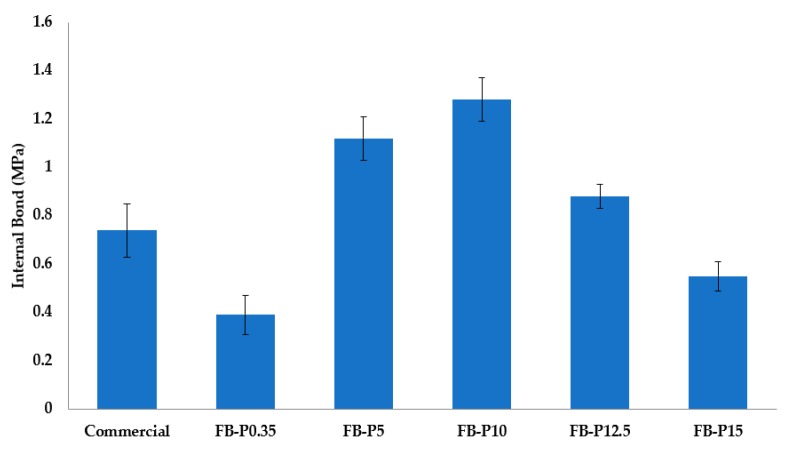
Mean internal bond (IB) of the produced all-lignocellulosic fiberboards.

Several research studies have already shown important effects of the pressing conditions (time, temperature and pressure) on fiberboard's mechanical properties [8,12,24]. In this paper, the effects of pressing pressure ranging from 0.35 MPa to 12.5 MPa on the mechanical properties of all-lignocellulosic fiberboards made from *Arundo* steam exploded fibers by means of wet process are presented. These levels were chosen on the basis of our own previous experiences in producing fiberboards with steam exploded fibers and also the literature review [11,13,24]. Table 2 shows the mechanical properties of various all-lignocellulosic fiberboards prepared at different pressing pressure, also illustrated in Figure 4, Figure 5 and Figure 6. 

The mechanical performances of fiberboards are usually expressed by the modulus of rupture (MOR) and the modulus of elasticity (MOE) [25,26]. They were analyzed together because they came from the same bending test. As shown in Figure 4 and Figure 5, the MOE and MOR of the commercial fiberboard were about 2670 and 41.7 MPa, respectively [27]. Overall, The MOE was increased by increasing the pressing pressure up to 12.5 MPa. Further increase in pressing pressure decreased MOE, especially for the fiberboards produced at 15 MPa. The highest value for MOE was obtained for the pressing pressure of 12.5 MPa, which was more than the double (7439 MPa) the stiffness required by the standard specifications. On the other hand, it is interesting to note that the MOE values found in this study were superior to some reported in earlier studies. For example, those of fiberboards made from corn biomass and cellulose nanofibers [26], or non-wood fibers with the addition of lignin [16,23]; or they were similar to the newly prepared binderless all-cellulose fiberboards from microfibrillated flax fibers [28]. 

For the modulus of rupture, Figure 5 revealed that MOR values of all-lignocellulosic fiberboards prepared at pressing pressure between 5 and 12.5 MPa, were comparable to the commercial board and in the same magnitude of the required MOR values by the standard specifications. It is interesting to note that the strength of fiberboards was increased as the pressing pressure was increased up to 12.5 MPa, after which this value was decreased. The lower and higher pressing pressure tested in this research work led to MOR values below the required standard specifications. However, the highest MOR value found in this research was superior to those of fiberboards made of alkaline hydrolyzed Kraft lignin and *Vitis vinifera*; and similar to that of manufactured fiberboards from purified Kraft lignin and *Vitis vinifera* fibers [16].

The internal bond (IB), also called tensile strength perpendicular to the faces, refers to the bonding strength between fibers which is important because it ensures that the boards will not delaminate during post-treatment [16]. The effect of pressing pressure on the IB is shown in Figure 6. For this property, the changes in the trend can be divided into two stages. In the first stage, the applied pressing pressure in the range of 0.35–10 MPa improved the internal bond. More specifically, fiberboards pressed only at 0.35 MPa showed the required IB strength by the standard specifications. What is even more interesting is that the increases in IB of the pressed all-lignocellulosic fiberboards at 5 MPa and 10 MPa were about 86.7% and 172%, respectively, compared to the commercial board. In addition, it is interesting to note that the maximum IB strength of about 1.28 MPa was already about three times superior to the commercial board. In the second stage, the pressing pressures were 12.5 and 15 MPa. In this stage the internal bond decreased with an increase in pressing pressure. The reduction in IB of the pressed all-lignocellulosic fiberboards at 12.5 and 15 MPa were of about 31% and 55%, respectively, compared to the maximum value found for fiberboard pressed at 10 MPa. These results suggested that a high pressing pressure leads to the deterioration of the mechanical performance of fiberboards. According to the European standard specifications, all prepared all-lignocellulosic fiberboards even at higher pressing pressure showed strength properties superior to those required for high density fiberboards. On the other hand, these values were above that of fiberboards made with 20% of Kraft lignin [16] and other values found in the literature [24,29,30]. This improvement in internal bond property indicated a good adhesion between fibers interfaces. 

Finally, in order to elucidate the effect of density on fiberboard’s mechanical properties, the calculation of specific mechanical properties is presented in Table 3. This was possible by measuring the density of the fiberboards. These densities were above 900 kg/m^3^ so the manufactured fiberboards are classified within the category of high density fiberboards according to the European Union standard (EN323). The results (Table 3) indicated that all the specific mechanical properties showed the same pattern as for the studied mechanical properties despite the removal of the density effect. Therefore, the obtained all-lignocellulosic fiberboards made with *Arundo* steam exploded fibers at the optimum pressing conditions have a higher specific internal bond, a more specific modulus of rupture and a more specific modulus of elasticity than the commercial board.

In order to explain these better mechanical properties of *Arundo* steam exploded fiberboards, it is necessary to better understand the bonding mechanism between fibers during pressing. It is well known that the values of mechanical properties depend on: (1) the bonding strength among fibers, and (2) individual fiber strength. In our case, better inter-fiber bonds were the main factor responsible for the improvement of bending strength, as the fibers used were of the same quality. These inter-fiber bonds are due mainly to: (i) hydrogen bonding between fibers, (ii) the condensation reaction in lignin polymer, (iii) the cross-linking reaction between lignin and polysaccharides, and (iv) the formation of covalent bonds between the constituents of lignocellulosic polymers [31,32,33,34,35,36]. 

The greater mechanical properties found in this study can be attributed to all the above mentioned inter-fiber bonds mechanisms. First, the formation of covalent bonds and hydrogen bonding between the lignocellulosic polymers, during both room temperature and hot pressing accompanied by the water loss, can occur since the fibers used are constituted by lignin, cellulose and hemicelluloses [16,24,31, 32,33,34,35]. As is well known, the covalent bonds result in intermolecular forces that are much stronger than those of hydrogen bonds. On the other hand, polysaccharides and lignin are polymeric materials consisting of various functional groups such as hydroxyl, carbonyl and carboxylic groups [37]. These functional groups can cause the condensation reaction in lignin polymer and the crosslinking reaction between lignin and polysaccharides under hot pressing conditions. In addition, fibers with lignin-rich surfaces from the applied steam explosion process can improve the mechanical properties of all-lignocellulosic fiberboards through the mechanical entanglement of the melted lignin molecules under pressure and temperature, possibly accompanied by the formation of covalent bonds [24,33,34]. In addition, this improvement can be explained by the reduction of void spaces between fibers which are due mainly to the increased pressing pressure and the homogenous distribution of the fibers [8,28]. 

## 3. Materials and Methods

### 3.1. Materials

The *Arundo donax* L. used in this study was obtained from the banks of the natural course of ravine in the municipality of Riudecanyes, province of Tarragona, Spain. The rods used were two years old and were chopped with a GA 100 chipper (Black and Decker, Towson, MD, USA), leaving pieces of 3–8 cm long and about 2 cm thick. The chopped material was stored in jute bags to obtain an equilibrium with environmental conditions up until the completion of the steam explosion treatment. 

### 3.2. Steam Explosion and Grinding

**Figure 7:**
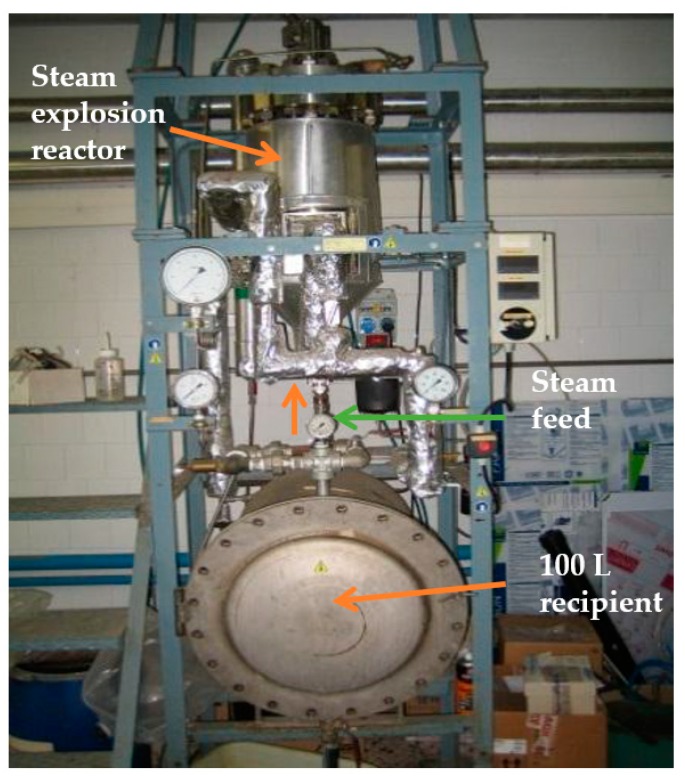
Steam explosion reactor.

The *Arundo donax* chips of about 800–1000 g dry basis per batch were fed into the steam explosion reactor shown in Figure 7. The reactor is a stainless steel, cylindrical batch-type reactor with a nominal capacity of 16 L, 45 bars of pressure, and a temperature of 250 °C [16]. A pneumatic valve connects the reactor to a 100 L recipient in which the pretreated material is collected after the flash expansion process. Steam is fed into the reactor at the bottom to facilitate the impregnation of the material.

The chips were then treated with saturated steam at a temperature of 210 °C and time of 9.5 mns. After the set time was reached, the chips were quickly depressurized into a 100 L recipient. These conditions were chosen based on our previous research experiences with other fibers and similar materials such as *Miscanthus sinensis* [12]. This treatment helps to defibrillate the material. The pulp obtained from this pre-treatment was washed with distilled water to rinse out the pulping liquor derived from the pre-treatment process; this liquor contains mainly extractives and hemicelluloses. Finally, the obtained pulp was air dried at room temperature until the moisture content was of around 12%, and stored in plastic bags for subsequent grinding and its chemical characterization. 

The pretreated pulps were ground and passed through a 4 mm sieve. Previous studies have shown that pulp milling increases the bonding area and thus improve the internal bond strength [11].

### 3.3. Fiberboard Production

**Figure 8:**
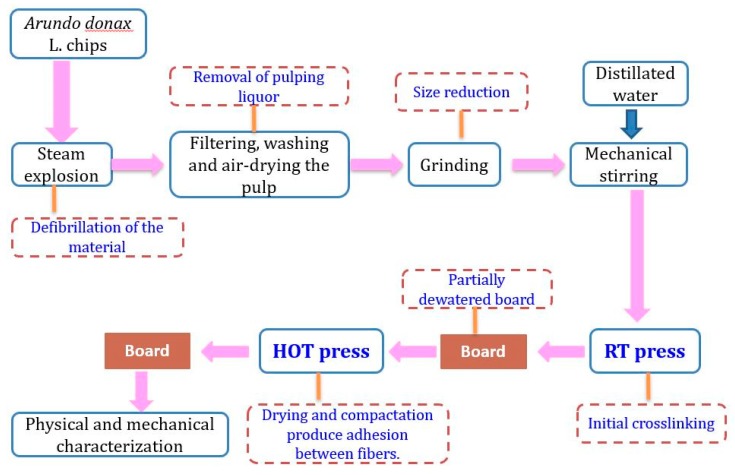
Experimental procedure of the production of all-lignocellulosic fiberboards.

**Figure 9:**
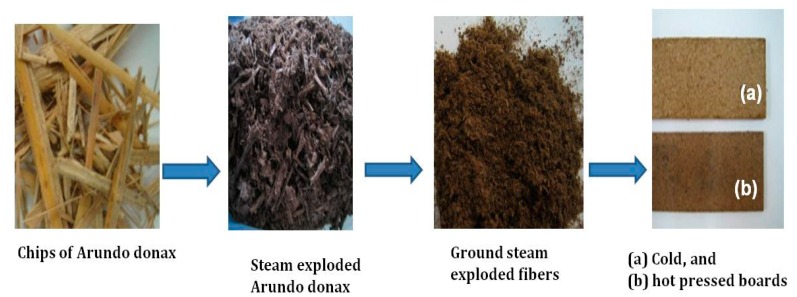
Scheme of the *Arundo donax* raw material and the final all-lignocellulosic fiberboard.

Different natural fibers from wood and non-wood materials with different morphological and chemical composition can be used for the production of all-lignocellulosic fiberboards. Here, the steam exploded *Arundo donax* fibers were used and the experimental procedure for the manufacturing of all-lignocellulosic fiberboard is illustrated in Figure 8. The first step involved cold forming and stabilization under constant temperature and relative humidity. This allowed the mold to be filled in a convenient way, leading towards improving the uniformity of the produced fiberboards. For this, 28.5 g of ground-pre-treated material was disintegrated in 185 mL distillated water by using a laboratory mechanical stirrer at 900 rpm for 5 min to ensure good fiber distribution. Then, the fibers suspension was introduced and distributed evenly into a mold, and cold pressed at 16 MPa in a room temperature (RTP) press (AN MEGA-30, Berriz, Spain), creating a preformed board. The cold pressing involved a filtering of the fibers suspension and partial dewatering in order to get the desired consistency of fibers for subsequent stabilization. Then, the fiberboards were conditioned in a climatic chamber (MMM group CLIMACELL 111, Planegg, Germany) at 20 °C and 65% relative humidity and kept in these conditions until achieving a constant weight. The initial step in the formation of interfiber interactions occurred when the samples were subjected to room temperature pressing pressure where removal of water took place. In the second step, the partially dewatered boards were hot pressed on a heated press (Servitec Polystat 300S, Wustermark, Germany) at 205 °C for 3.5 min, followed by a 1 min decompression to allow the steam to be released from the test probe avoiding bubble formation, and finally by a second pressing stage of 3.25 min; the total pressing time was 7.5 min. Different all-lignocellulosic fiberboards were prepared at different pressing pressure ranging from 0.35 MPa to 15 MPa. To facilitate the drying process and to avoid bubbles formation during hot pressing, a steam evacuation mesh, a pre-formed board, a cover, and finally the mold piston were then placed inside the mold in the corresponding order. The high pressing temperature of 205 °C was selected to favour the possible effects of lignin plasticization on the self-bonding mechanism. In order to further understand the procedure, Figure 9 illustrates the scheme of the raw materials and the final boards pressed with both cold and hot press machines.

### 3.4. Chemical Characterization

The chemical composition of the original raw material and pulp were determined according to standard methods from the American Society for Testing and Materials (ASTM). The chemical properties analyzed were moisture content (ASTM E871-82, 2006), ash content (ASTM D1102-84, 2001), and Klason lignin (KL) (ASTM D1106-96, 2007). The carbohydrates from the Klason lignin hydrolysis test were analyzed using HPLC [38] to determine cellulose and hemicelluloses content. Acid-soluble lignin (ASL) was also determined using the UV absorption method [39]. Triplicate samples were used to determine the chemical properties.

### 3.5. Physical and Mechanical Characterization

The physical and mechanical properties of fiberboards were determined according to the European standards. Boards were conditioned at 20 °C and 65% RH before any physical or mechanical tests were conducted, and the dimensions of the test pieces were determined on the basis of the EN325 standard (EN325). The mechanical properties measured were: internal bond (IB) (EN319); modulus of elasticity (MOE) and modulus of rupture (MOR) (EN310). Dimensional stability was characterized by measuring: thickness swelling (TS) and water absorption (WA) (EN317). Additionally, the density of the boards was determined (EN323). The European standards for these properties are as follows: Density > 900 kg/m^3^, MOR ≥ 40MPa, MOE ≥ 3000MPa, IB ≥ 0.7MPa, WA ≤ 30%, and TS ≤ 20%.

## 4. Conclusions

It was possible to produce all-lignocellulosic fiberboards that meet the relevant standard specifications by means of wet processing using *Arundo donax* as raw material. The *Arundo donax* raw material exhibited relatively high cellulose and moderate lignin percentages, which are desirable for all-lignocellulosic fiberboards. The selected steam explosion condition has decreased hemicelluloses and extractives, leading to fibers with high hydrophobicity and lignin rich-surface fibers, that improve both physical and mechanical properties of the boards. 

The influence of pressing pressure on the physical and mechanical properties of the boards was evaluated and it was concluded that increasing pressing pressure had a positive effect on these tested properties. The densities of fiberboards were increased by increasing the pressing pressure leading to a dense material with fewer voids. The less voids content responsible for more fiber-fiber interactions and the self-bonding mechanism of steam exploded fibers can explain the good mechanical properties obtained of all-lignocellulosic fiberboards. The optimum IB, MOE, MOR, WA and TS were 1.28 MPa, 7439 MPa, 40.4 MPa, 17.6% and 13.3%, respectively. Therefore, the prepared all-lignocellulosic fiberboards were of very good quality and by far satisfy the requirements of the relevant standard specifications. To sum up, *Arundo* steam exploded fibers can be used as a source of fiber suitable for the manufacturing of fiberboards. 

## Figures and Tables

**Table 1:** Results of chemical analysis of *Arundo donax* raw material and its steam exploded pulp compared with other lignocellulosic materials.

Raw Materials	Ash-C (%)	Extract (%)	Lignin (%)	Cellulose (%)	HMC (%)	H-Cell (%)
*Arundo donax*	3.8 ± 0.5	9.3 ± 0.3	22.4 ± 0.2	43.1 ± 0.5	21.9 ± 0.2	65 ± 0.5
*Arundo donax pulp*	1.2 ± 0.2	5.9 ± 0.5	29 ± 0.9	55.4 ± 0.9	5.2 ± 0.8	-
*Arundo biomass* [2,6]	4.2–6.1	11.2–21.6	19.2–24.3	29.2–39.1	14.5–32.0	-
*Miscanthus sinensis* [12]	0.7	4.7	19.9	42.6	21.1	-
*Vitis vinifera* [16]	3.70	13.30	24	43.60	19.10	62.70
*Pine pinaster* [15]	0.54	--	26.20	55.90	13.70	69.60
*Eucalyptus globulus* [15]	0.57	--	20.00	52.80	27.70	80.50

Ash-C: Ash content; Extract: extractives; HMC: hemicelluloses; H-Cell: holocellulose.

**Table 2:** Results of physical and mechanical properties of all-lignocellulosic fiberboards.

Samples	d (kg/m^3^)	WA (%)	TS (%)	MOR (MPa)	MOE (MPa)	IB (MPa)
Commercial [27]	893 ± 19	76.0 ± 5.0	66.0 ± 4.0	41.7 ± 1.6	2670 ± 110	0.74 ± 0.11
FB-P0.35	1042 ± 38	44.1 ± 5.0	22.5 ± 5.0	13.9 ± 0.7	2955 ± 100	0.39 ± 0.08
FB-P5	1138 ± 24	25.0 ± 5.0	19.5 ± 5.0	38.5 ± 0.4	6516 ± 190	1.12 ± 0.09
FB-P10	1202 ± 14	20.3 ± 4.0	13.3 ± 3.0	40.5 ± 0.4	7256 ± 140	1.28 ± 0.09
FB-P12.5	1222 ± 12	17.6 ± 2.0	13.6 ± 2.0	40.4 ± 0.3	7439 ± 160	0.88 ± 0.05
FB-P15	1231 ± 18	17.7 ± 2.0	13.9 ± 2.0	23.1 ± 0.8	5598 ± 150	0.55 ± 0.06

d: density, WA: water absorption, TS: thickness swelling, MOR: modulus of rupture, MOE: modulus of elasticity, IB: internal bond. FB-P5: fiberboard pressed at 5 MPa.

**Table 3:** Specific mechanical properties of all-lignocellulosic fiberboards.

Samples	Specific-MOR (MPa·m^3^/kg)	Specific-MOE (MPa·m^3^/kg)	Specific-IB (MPa·m^3^/kg)
Commercial	4.67 × 10^−2^	2.99	5.26 × 10^−4^
FB-P0.35	1.33 × 10^−2^	2.84	3.74 × 10^−4^
FB-P5	3.21 × 10^−2^	5.73	9.84 × 10^−4^
FB-P10	3.37 × 10^−2^	6.04	10.65 × 10^−4^
FB-P12.5	3.31 × 10^−2^	6.09	7.20 × 10^−4^
FB-P15	1.88 × 10^−2^	4.55	4.47 × 10^−4^
